# Development of a machine learning-based predictive nomogram for screening children with juvenile idiopathic arthritis: a pseudo-longitudinal study of 223,195 children in the United States

**DOI:** 10.3389/fpubh.2025.1531764

**Published:** 2025-05-29

**Authors:** Yu-Sheng Lee, Kira Gor, Matthew Evan Sprong, Junu Shrestha, Xueli Huang, Heaven Hollender

**Affiliations:** ^1^School of Integrated Sciences, Sustainability, and Public Health, College of Health, Science, and Technology, University of Illinois Springfield, Springfield, IL, United States; ^2^Department of Addictions Studies and Behavioral Health, College of Health and Human Services, Governors State University, University Park, IL, United States; ^3^Department of Computer Science, College of Health, Science, and Technology, University of Illinois Springfield, Springfield, IL, United States; ^4^School of Health and Human Sciences, Indiana University Indianapolis, Indianapolis, IN, United States

**Keywords:** juvenile idiopathic arthritis, pediatric arthritis, pediatric joint inflammation, chronic rheumatology, LASSO, machine learning, nomogram, NSCH

## Abstract

**Background:**

Juvenile idiopathic arthritis (JIA) is a prevalent chronic rheumatological condition in children, with reported prevalence ranging from 12. 8 to 45 per 100,000 and incidence rates from 7.8 to 8.3 per 100,000 person-years. The diagnosis of JIA can be challenging due to its symptoms, such as joint pain and swelling, which can be similar to other conditions (e.g., joint pain can be associated with growth in children and adolescents).

**Methods:**

The National Survey of Children's Health (NSCH) database (2016–2021) of the United States was used in the current study. The NSCH database is funded by the Health Resources and Services Administration and Child Health Bureau and surveyed in all 50 states plus the District of Columbia. A total of 223,195 children aged 0 to 17 were analyzed in this study. A least absolute shrinkage and selection operator (LASSO) logistic regression and stepwise logistic regression were used to select the predictors, which were used to create the nomograms to predict JIA.

**Results:**

A total of 555 (248.7 per 100,000) JIA cases were reported in the NSCH. In the LASSO model, the receiver operating characteristic curve demonstrated excellent discrimination, with an area under the curve (AUC) of 0.9002 in the training set and 0.8639 in the validation set. Of the 16 variables selected by LASSO, 13 overlapped with those from the stepwise model. The regression achieved an AUC of 0.9130 in the training set and 0.8798 in the validation set. Sensitivity, specificity, and accuracy were 79.1%, 90.2%, and 90.2% in the training set, and 69.0%, 90.9%, and 90.8% in the validation set.

**Discussion:**

Using two well-validated predictor models, we developed nomograms for the early prediction of JIA in children based on the NSCH database. The tools are also available for parents and health professionals to utilize these nomograms. Our easy-to-use nomograms are not intended to replace the standard diagnostic methods. Still, they are designed to assist parents, clinicians, and researchers in better-estimating children's potential risk of JIA. We advise individuals utilizing our nomogram model to be mindful of potential pre-existing selection biases that may affect referrals and diagnoses.

## 1 Introduction

Juvenile idiopathic arthritis (JIA) is a prevalent chronic rheumatological condition in children (1–3), with reported prevalence ranging from 12.8 to 45 per 100,000 and incidence rates from 7.8 to 8.3 per 100,000 person-years (4–9). This disease can significantly impact the quality of life, physical function, and psychological wellbeing of children and their families (10). The financial cost of JIA can be high. A systematic review observed that annual costs for JIA can vary significantly, ranging from $310 up to $44,832 per patient. This variation is largely influenced by several factors, including the country where treatment is administered, the level of disease activity, the specific subtype of JIA, and whether biological therapies are utilized (10).

JIA is a general term for unexplained idiopathic inflammatory arthritis affecting children younger than 16 years of age and lasting 6 weeks or longer (3). It is categorized into seven subtypes: oligoarticular JIA, seropositive polyarticular JIA, seronegative polyarticular JIA, systemic-onset JIA, enthesitis-related arthritis, juvenile psoriatic arthritis, and undifferentiated JIA (11). The cause of JIA is unknown and highly unpredictable (12) but it is considered a combination of genetic and environmental factors (1, 13). For example, smoking during pregnancy is an environmental risk factor for JIA, while breastfeeding and having siblings may reduce the risk (14). The distribution of JIA based on onset age is bimodal, with peaks at <4 years, as stated, and a second peak in early adolescence (15, 16). Some forms of JIA are more common in girls than in boys (16–18). It can also lead to severe complications such as growth problems, muscle weakness and loss, and eye inflammation (16, 19–22).

The diagnosis of JIA can be challenging due to its symptoms, such as joint pain, swelling, stiffness, and damage (16), which can be similar to those of other conditions, including infections, injuries, or other forms of arthritis (23, 24). To diagnose JIA, medical professionals must rule out the conditions of joint symptoms above based on clinical evaluation, medical history, and a musculoskeletal examination (3, 25, 26). Moreover, the symptoms and clinical features can vary significantly from child to child, making it necessary to consider various differential diagnoses carefully. Currently, no single doctor or specific test, such as a blood test or imaging study, is available to diagnose JIA (3, 25, 27). This disease can be a chronic condition that evolves. Children with JIA may have intermittent or unrecognized symptoms, leading to delayed diagnosis (28). A study found that 42% of the patients had more than 3 months (29) of delay from symptom onset to physician diagnosis. Diagnosing JIA often requires the expertise of a pediatric rheumatologist (25). Not all healthcare providers have the necessary experience or training to make a JIA diagnosis, which can lead to delays in receiving appropriate care (30).

Early detection and treatment of JIA offer several significant benefits for children who are affected (31). It allows for the timely initiation of appropriate therapy, which helps to control the disease better, reduce inflammation, and minimize damage to the joints. Early detection and treatment can also significantly improve a child's overall quality of life (22, 26, 31). Children can continue to engage in school, sports, and social activities with less disruption due to their condition (32). In addition, JIA can lead to joint damage over time if left untreated. Early intervention can help preserve joint function and mobility, preventing long-term disability and deformities (22, 24, 26).

This study aims to develop a nomogram to assist in predicting the likelihood of JIA diagnoses in children, i.e., to predict which children go on to develop JIA from an earlier time point. A nomogram is a visual statistical instrument for physicians to estimate the individual probability of disease development or death (33). Although other assessments are all methods that assist in correctly diagnosing JIA, the nomogram will contribute to the pre-screening process for further confirmation that additional evaluation is required. The following research questions guided the current study:

(1) Which demographic and clinical factors predict the diagnosis of JIA?
(2) Are the identified nomograms valid in estimating the individualized probability of JIA in a given child?

## 2 Materials and methods

### 2.1 Source of data

The National Survey of Children's Health (NSCH) data (2016–2021) was used in the current study. The NSCH database is funded by the United States (U.S.) Health Resources and Services Administration and Child Health Bureau to collect physical and mental health, access to quality health care, and the child's family, neighborhood, school, and social context information (9, 34, 35) of children ages 0 to 17 surveyed across all 50 U.S. states plus the District of Columbia, a federal district that is not part of any state but is included as a separate population source. The number of children surveyed in each state/district is shown in Supplementary Table 1. The NSCH, conducted by the U.S. Census Bureau on behalf of the Health Resources and Services Administration's Maternal and Child Health Bureau, was carried out both online and by mail if there were one or more children ages 0 to 17 living in the household. Instructions for accessing the online survey were sent to randomly selected households from across the U.S. Following two reminder letters and postcard notifications encouraging online participation, households that still hadn't accessed the survey were provided with a paper screening questionnaire (36). Additional information about the sampling and administration process, survey methodology, nonresponse bias analysis, and other pertinent information can be found on the survey's website at https://www.childhealthdata.org/learn-about-the-nsch/NSCH.

### 2.2 Study population

The 2016 NSCH was conducted from June 2016 through February 2017 (139,923 households screened; 67,047 were eligible; 50,212 child-level topical interviews were completed nationally); the 2017 NSCH was conducted between August 2017 and February 2018 (59,135 households screened; 29,343 were eligible; 21,599 child-level topical interviews were completed nationally); the 2018 NSCH was conducted between June 2018 and January 2019 (176,052 households screened; 38,140 were eligible; 30,530 child-level topical interviews were completed nationally); the 2019 NSCH was conducted between June 2019 and January 2020 (180,000 households screened; 35,760 were eligible; 29,433 child-level topical interviews were completed nationally); the 2020 NSCH was conducted between June 2020 and January 2021 (93,840 households screened; 51,107 were eligible; 42,777 child-level topical interviews were completed nationally); and the 2021 NSCH was conducted between July 2021 and January 2022 (106,000 households screened; 62,010 were eligible; 50,892 child-level topical interviews were completed nationally). The response rates were between 86% and 92% (36). Since 2016, NSCH data can be combined to increase the analytic sample size and can investigate the time-series trend with multiple years of non-overlapping sampling data (37). The NSCH compared response rates across various demographic and socioeconomic subgroups to highlight disparities. The analysis examined the effectiveness of weighting adjustments to reduce nonresponse bias. The weighting process for interviewed children started with a base weight for each sampled household, followed by a nonresponse adjustment for the screener. Eligible children were then adjusted using a Child-Level Screener Factor and a within-household subsampling factor. A nonresponse adjustment for topical issues was applied, followed by a ranking adjustment to demographic controls and trimming of extreme weights if necessary. Although findings indicated some differences between respondents and nonrespondents, the weighting adjustments were generally effective in minimizing the nonresponse bias and enhancing the survey's representativeness (34, 38–40). Additional information about the sampling and administration process, survey methodology, nonresponse bias analysis, and other pertinent information can be found on the survey's website (34). The NSCH is a public database that does not contain any personal identifiers. With the approval of the Institutional Review Board (IRB) from the primary author's university (University of Illinois at Springfield IRB approval number 25-006), we conducted a pseudo-longitudinal (repeated cross-sectional) study (41–43) in accordance with the Strengthening the Reporting of Observational Studies in Epidemiology (STROBE) guidelines to ensure methodological rigor and transparency in reporting. Children who did not provide the information on JIA-related questions during the surveys and had missing values in the dataset (*n* = 2,248) were excluded from the study analysis. The definition of JIA will be presented in the Section 2.3.

### 2.3 Outcome

Throughout the six waves of the survey, parents were asked the same following questions: “Has a doctor or other health care provider ever told you that this child has arthritis?” (Yes vs. No) and “Does this child currently have arthritis?” (Does not currently have the condition vs. Current condition) If parents responded “Yes” to the first question and “current condition” to the second question, a child was defined as having JIA. Others were defined as non-JIA.

### 2.4 Predictors

A total of 22 potential predictors from the NSCH database at the time of survey included in the model selection were the child's age, sex, race/ethnicity, maternal age at delivery, premature birth, whether low birth weight, months of breastfeeding, age/sex-standardized body mass index (BMI), having a genetic or inherited condition identified through a blood test, tobacco use in household, allergy to food, drug, or insect, asthma, Type 1 Diabetes, heart condition, depression, anxiety, household's ability to afford the food you need during the past 12 months, frequency of physical activity, chronic physical pain (including headaches or other back or body pain in the past 12 months), difficulty with eating or swallowing in the past 12 months, adequacy of current insurance coverage, and child with a personal doctor or nurse.

### 2.5 Statistical analysis

Between-group comparisons were conducted using a t-test for continuous variables and a Mann-Whitney U test when the normality assumption was unmet. For categorical variables, differences were estimated using the Chi-square test or Fisher's Exact test.

#### 2.5.1 Predictor variable selection

We used a least absolute shrinkage and selection operator (LASSO) logistic regression and a stepwise logistic regression to select the predictors for JIA. In terms of statistical regressions, both linear and logistic regression aim to estimate the relationship between predictor variables and an outcome variable by fitting a model that best explains the observed data. Traditional regression methods find coefficient values that maximize the likelihood of the data under the assumed model. However, when there are numerous predictors or when some variables are highly correlated, researchers encounter overfitting and poor generalization issues. LASSO regression harnesses regularization to address these issues by penalizing model complexity. Instead of merely maximizing likelihood as in ordinary regression, LASSO method optimizes a penalized likelihood function that includes an extra constraint on the coefficients' size. Specifically, it maximizes the usual likelihood function while summing an L1 penalty to it in the form of $\lambda \sum_{j=1}^{p}|\beta_{j}|$, where λ is a regulating/tuning parameter. The effect of this penalty not only shrinks the coefficients toward zero but also, importantly, forces some of them to be exactly zero, i.e., this enables LASSO regression to prevent overfitting issues and perform the variable selection. The λ controls the balance between model complexity and predictive accuracy; when λ is small (close to zero), the penalty is minimal, making LASSO behave similarly to ordinary regression; when λ increases, more coefficients shrink toward zero, simplifying the model by reducing the number of predictors. This trade-off is especially useful in cases with many variables but limited observations, highlighting LASSO's ability to select the most relevant variables while estimating their effects (44). Variable selection in LASSO regression refers to automatically selecting only the predictors that are significant and excluding irrelevant predictors from the model. This method identifies the optimal value for the regularization parameter λ. We used the *glmnet* package in R to select variables based on a k-fold cross-validation approach, which divides the dataset into k subsets, or “folds.” Each fold then serves as a validation set, while the remaining k-1 folds form the training set. This process is repeated k times, ensuring that each fold is used for validation exactly once. During each iteration, the model is trained on the training set with a specific λ value and evaluated on the validation set. This evaluation typically focuses on metrics such as classification accuracy, mean squared error of prediction, or deviance. Because we used LASSO logistic regression, the deviance metrics were evaluated. By averaging these performance metrics across all k folds, an estimate of the model's effectiveness was obtained for that specific λ. This procedure is repeated across a range of λ values, generating a performance profile for each. By comparing these profiles, determine the λ that minimizes the average validation error, signifying the optimal balance between model fit and complexity (44). Although using the maximum λ value, one standard error, improves model parsimony and lowers the risk of overfitting (45), using the minimum λ value balances model complexity and prediction accuracy (46–49). As a result, we selected the optimal minimum λ value through cross-validation. To cross-validate the LASSO logistic models, the data was split randomly into the training (70%) and validation (30%) sets using the SAS SURVEYSELECT procedure.

The stepwise logistic regression was used as the second method to identify significant predictors of JIA with the SAS LOGISTIC procedure, which, by default, starts with an empty model with no predictors. At each step, predictors are either added or removed based on statistical criteria (bidirectional selection with a significant entry level of 0.15 and an exit level of 0.15, i.e., a variable has to be significant at the 0.15 level before it can be entered into the model, and a variable in the model has to be significant at the 0.15 level for it to remain in the model) to find the model that minimizes the Akaike Information Criterion (50). These statistical criteria are commonly used in stepwise selection methods to allow potentially important variables to enter the model while controlling for overfitting (51, 52). We chose 0.15 other than the conventional 0.05 to reduce the risk of prematurely excluding variables that may have meaningful contributions when considered in combination with others.

After the variable selections, we used those variables selected by LASSO and stepwise logistic regressions to create two nomograms for predicting the occurrence of JIA, utilizing the R *rms* package. Additionally, we applied these selected variables in the multivariable logistic regressions to estimate the odds ratios (OR) along with the 95% confidence intervals (CI) for JIA.

#### 2.5.2 Model performance

The prediction model selected from the training set was applied to the validation set to validate and evaluate the prediction efficacy. The receiver operating characteristic (ROC) curve and the area under the curve (AUC) were estimated to verify the discrimination performance in the training and validation sets. To ensure model stability and calibration, we performed 1000 bootstrap resamples. In each resample, LASSO was used to select a subset of predictors while shrinking others to zero. The final set of variables included those consistently selected across a high proportion of bootstrap samples, with their coefficients averaged from nonzero estimates. Calibration plots were created using the R *rms* package, incorporating bootstrapped estimates to evaluate the agreement between predicted and observed probabilities. The Decision Curve Analysis (DCA) was used to estimate the clinical effectiveness of the model for JIA patients by the R *rmda* package with 1,000 bootstrapping re-samples. DCA can be used to estimate the net benefits of a model based on the difference between the number of true-positive and false-positive results to assess the clinical usefulness of the identified models. It evaluates whether a particular model or test is beneficial for making clinical decisions by considering the balance between the benefits and harms of using it. If the DCA curve is consistently above the horizontal reference line (test none), the model is beneficial for making clinical decisions across a range of probability thresholds. If the DCA curve is consistently above the reference line (test all), it suggests that the test adds value and avoids unnecessary interventions.

#### 2.5.3 Missing values

For variables (e.g., age, race, family economic status, or health conditions) with missing values, we performed listwise deletions in the R package, meaning the entire observation was removed from the analysis. In SAS, the LOGISTIC procedure automatically excluded observations with missing values from the analyses.

All analyses were performed in SAS package version 9.4 (SAS Institute Inc., NC) and R package version 4.2.2.

## 3 Results

A total of 223,195 children were included in the analysis. This included 555 (248.7 per 100,000) JIA cases. The mean age was 9.14 (standard deviation [SD] = 5.26), which was slightly lower than those children who did not provide the information on JIA diagnosis with a mean age of 10.00 (SD = 5.14). The detailed comparison is shown in Supplementary Table 2. Of the 223,195 children, 51.8% were boys, 68.3% were White, followed by Hispanic 12.4% and Black 6.3%; of the 2248 children with missing JIA values, 50.7% were boys, 60.9% were White, followed by Hispanic 14.4% and Black 10.9%.

Table 1 showed the demographic and clinical characteristics of the study population for both the training and validation sets, revealing no significant differences between the two groups. Table 2 demonstrated the univariate analysis of the 22 potential predictors of JIA, indicating that all 22 variables were statistically associated with JIA. Specifically, girls were more likely to report having JIA than boys. Additionally, children with JIA, compared to those without, had a lower maternal age at delivery (29.2 vs. 30.2), a higher prevalence of premature births (17.7% vs. 10.8%), of low birth weight (14.9% vs. 8.4%), of overweight or obesity (33.6% vs. 13.7%), of household tobacco use (19.6% vs. 13.4%), of asthma (10.3% vs. 2.2%), and of heart conditions (6.7% vs. 1.4%).

**Table 1:** Demographic and clinical characteristics of the NSCH study population, stratified by training and validation groups.

**Characteristics**	**Validation group**	**Training group**	**Total**	***p*-value**
	***N*** **(%)**	***N*** **(%)**	***N*** **(%)**	
**Juvenile idiopathic arthritis**				0.6115
No	66,796 (99.74)	155,844 (99.75)	222,640 (99.83)	
Yes	172 (0.26)	383 (0.25)	555 (0.25)	
**Child's age when survey [mean(SD)]**	9.18 (5.26)	9.14 (5.26)	9.15 (5.26)	0.1106
**Sex**				0.4724
Boy	34,923 (51.6)	81,749 (51.8)	116,672 (51.7)	
Girl	32,709 (48.4)	76,062 (48.2)	108,771 (48.3)	
**Race**				0.1755
Hispanic	8,540 (12.7)	19,315 (12.3)	27,855 (12.4)	
White, non-Hispanic	45,964 (68.1)	107,552 (68.3)	153,516 (68.3)	
Black, non-Hispanic	4,240 (6.3)	10,075 (6.4)	14,315 (6.4)	
Asian, non-Hispanic	3,535 (5.2)	8,356 (5.3)	11,891 (5.3)	
American Indian or Alaska Native Non-Hispanic	408 (0.6)	940 (0.6)	1,348 (0.6)	
Others	4,766 (7.1)	11,209 (7.1)	15,975 (7.1)	
**Maternal age at delivery [Mean(SD)]**	30.18 (5.79)	30.17 (5.78)	30.17 (5.78)	0.8316
**Premature birth (Yes)**	7,196 (10.8)	16,862 (10.8)	24,058 (10.8)	0.7529
**Low birth weight**				0.6032
No	59,089 (91.5)	137,932 (91.6)	197,021 (91.6)	
Low birth weight	4,637 (7.2)	10,684 (7.1)	15,321 (7.1)	
Very low birth weight	826 (1.3)	1,984 (1.3)	2,810 (1.3)	
**Months of breastfeeding**				0.2780
6 months or longer, or still breastfeeding	10,625 (15.8)	25,200 (16.1)	35,825 (16.0)	
Children age 6–17 years	46,685 (69.5)	108,493 (69.3)	155,178 (69.3)	
<6 months	9,823 (14.6)	22,972 (14.7)	32,795 (14.7)	
**BMI**				0.0917
Normal weight	21,040 (31.8)	49,097 (31.8)	70,137 (31.8)	
Children age 0–9 years, BMI not measured	33,873 (51.1)	79,660 (51.5)	113,533 (51.3)	
Underweight	2,041 (3.1)	4,725 (3.1)	6,766 (3.1)	
Overweight or obese	9,288 (14.0)	21,083 (13.6)	30,371 (13.8)	
**Tobacco use in household**				0.1024
No one smokes in the household	58,399 (86.3)	136,794 (86.7)	195,193 (86.6)	
Someone smokes, not inside the house	8,102 (12.0)	18,433 (11.7)	26,535 (11.8)	
Someone smokes inside the house	1,131 (1.7)	2,584 (1.6)	3,715 (1.6)	
**Child's household food insecurity**				0.9119
Always afford to eat good nutritious meals	49,427 (74.8)	115,605 (74.9)	165,032 (74.9)	
Always afford enough to eat but not always the kinds of food we should eat	14,335 (21.7)	33,263 (21.6)	47,598 (21.6)	
Sometimes could not afford enough to eat	1,980 (3.0)	4,617 (3.0)	6,597 (3.0)	
Often could not afford enough to eat	357 (0.5)	844 (0.5)	1,201 (0.5)	
**Adequacy of current insurance coverage**				0.3187
Adequate	46,188 (68.7)	107,535 (68.5)	153,723 (68.6)	
Not adequate	18,217 (27.1)	42,453 (27.1)	60,670 (27.1)	
Uninsured	2,860 (4.3)	6,893 (4.4)	9,753 (4.4)	
**Physical activity (PA)**				0.4178
Everyday	20,947 (31.3)	49,318 (31.6)	70,265 (31.5)	
Children age 0–5 years, PA not measured	4,356 (6.5)	10,032 (6.4)	14,388 (6.4)	
4–6 days	18,111 (27.1)	41,751 (26.7)	59,862 (26.8)	
1–3 days	13,843 (20.7)	32,429 (20.8)	46,272 (20.7)	
0 day	9,705 (14.5)	22,771 (14.6)	32,476 (14.6)	
**Children with a personal doctor or nurse (Yes)**	51,632 (76.7)	120,418 (76.7)	172,050 (76.7)	0.9516
**Children's health condition (Yes)**				
Anxiety	6,102 (10.5)	14,132 (10.5)	20,234 (10.5)	0.6813
Allergy to food, drug, or insect	5,883 (8.7)	13,840 (8.8)	19,723 (8.8)	0.5680
Asthma	1,444 (2.2)	3,432 (2.2)	4,876 (2.2)	0.5468
Chronic physical pain in the past 12 months	4,361 (6.5)	9,997 (6.4)	14,358 (6.4)	0.3297
Depression	2,687 (4.6)	6,166 (4.6)	8,853 (4.6)	0.4999
Type 1 Diabetes	282 (0.4)	640 (0.4)	922 (0.4)	0.6947
Difficulty with eating or swallowing in the past 12 months	936 (1.4)	2,329 (1.5)	3,265 (1.5)	0.0944
Genetic or inherited condition	2,635 (3.9)	5,967 (3.8)	8,602 (3.8)	0.1985
Heart condition	913 (1.4)	2,172 (1.4)	3,085 (1.4)	0.6198

The table presents key variables including age, sex, race/ethnicity distribution, BMI, maternal age at delivery, length of breastfeed, and health conditions (e.g., anxiety, depression, asthma, allergy to food, drug, or insect, diabetes). Continuous variables are reported as mean ± standard deviation, while categorical variables are presented as N and %. Statistical comparisons between the training and validation groups were performed using appropriate tests (e.g., t-tests, Mann-Whitney U, chi-square, or Fisher exact test).

**Table 2:** Demographic and clinical characteristics of the NSCH study population, stratified by children with juvenile idiopathic arthritis (JIA) and without JIA.

**Characteristics**	**Non-JIA**	**JIA**	***p*-value**
	***N*** **(%)**	***N*** **(%)**	
**Child's age when survey [mean(SD)]**	9.13 (5.26)	13.29 (3.81)	<0.0001^*^
**Sex**			<0.0001^*^
Boy	115,318 (51.8)	215 (38.7)	
Girl	107,322 (48.2)	340 (61.3)	
**Race**			<0.0001^*^
Hispanic	27,478 (12.4)	55 (9.9)	
White, non-Hispanic	151,755 (68.3)	397 (71.7)	
Black, non-Hispanic	14,018 (6.3)	53 (9.6)	
Asian, non-Hispanic	11,719 (5.3)	8 (1.4)	
American Indian or Alaska Native Non-Hispanic	1,325 (0.6)	6 (1.1)	
Others	15,812 (7.1)	35 (6.3)	
**Maternal age at delivery [mean(SD)]**	30.17 (5.78)	29.17 (6.32)	<0.0001^*^
**Premature birth (Yes)**	23,751 (10.8)	97 (17.7)	<0.0001^*^
**Low birth weight**			<0.0001^*^
No	194,701 (91.6)	444 (85.1)	
Low birth weight	15,123 (7.1)	57 (10.9)	
Very low birth weight	2,748 (1.3)	21 (4.0)	
**Months of breastfeeding**			<0.0001^*^
6 months or longer, or still breastfeeding	152,969 (69.2)	520 (94.0)	
Children age 6–17 years	32,498 (14.7)	19 (3.5)	
Less than 6 months	35,557 (16.1)	14 (2.5)	
**BMI**			<0.0001^*^
Normal weight	112,474 (51.6)	90 (17.0)	
Children age 0–9 years, BMI not measured	6,668 (3.1)	31 (5.9)	
Underweight	69,167 (31.7)	231 (43.6)	
Overweight or obese	29,831 (13.7)	178 (33.6)	
**Tobacco use in household**			<0.0001^*^
No one smokes in the household	192,851 (86.6)	446 (80.4)	
Someone smokes, not inside the house	26,153 (11.8)	81 (14.6)	
Someone smokes inside the house	3,636 (1.6)	28 (5.0)	
**Child's household food insecurity**			<0.0001^*^
Always afford to eat good nutritious meals	163,231 (75.0)	291 (54.1)	
Always afford enough to eat but not always the kinds of food we should eat	46,869 (21.5)	181 (33.6)	
Sometimes could not afford enough to eat	6,471 (3.0)	52 (9.7)	
Often could not afford enough to eat	1,168 (0.5)	14 (2.6)	
**Adequacy of current insurance coverage**			<0.0001^*^
Adequate	151,969 (68.6)	295 (53.9)	
Not adequate	59,826 (27.0)	225 (41.1)	
Uninsured	9,590 (4.3)	27 (5.0)	
**Physical activity (PA)**			<0.0001^*^
Everyday	32,008 (14.5)	74 (13.7)	
Children age 0-5 years, PA not measured	69,671 (31.6)	35 (6.5)	
4–6 days	45,694 (20.7)	133 (24.5)	
1–3 days	59,025 (26.8)	200 (36.9)	
0 day	14,131 (6.4)	100 (18.5)	
**Children with a personal doctor or nurse (Yes)**	170,046 (76.8)	471 (86.1)	<0.0001^*^
**Children's health condition (Yes)**			
Anxiety	19,878 (10.5)	200 (36.6)	<0.0001^*^
Allergy to food, drug, or insect	19,505 (8.8)	156 (28.5)	<0.0001^*^
Asthma	4,789 (2.2)	56 (10.3)	<0.0001^*^
Chronic physical pain in the past 12 months	13,855 (6.3)	364 (66.2)	<0.0001^*^
Depression	8,643 (4.5)	117 (21.6)	<0.0001^*^
Type 1 Diabetes	893 (0.4)	16 (2.9)	<0.0001^*^
Difficulty with eating or swallowing in the past 12 months	3,181 (1.4)	58 (10.6)	<0.0001^*^
Genetic or inherited condition	8,412 (3.8)	126 (23.1)	<0.0001^*^
Heart condition	3,031 (1.4)	37 (6.7)	<0.0001^*^

* p < 0.05.

The table presents key variables including age, sex, race/ethnicity distribution, BMI, maternal age at delivery, length of breastfeed, and health conditions (e.g., anxiety, depression, asthma, allergy to food, drug, or insect, diabetes). Continuous variables are reported as mean ± standard deviation, while categorical variables are presented as N and %. Statistical comparisons between the children with and without JIA were performed using appropriate tests (e.g., t-tests, Mann-Whitney U, chi-square, or Fisher exact test).

### 3.1 Nomogram variables derived from LASSO logistic regression

**Figure 1:**
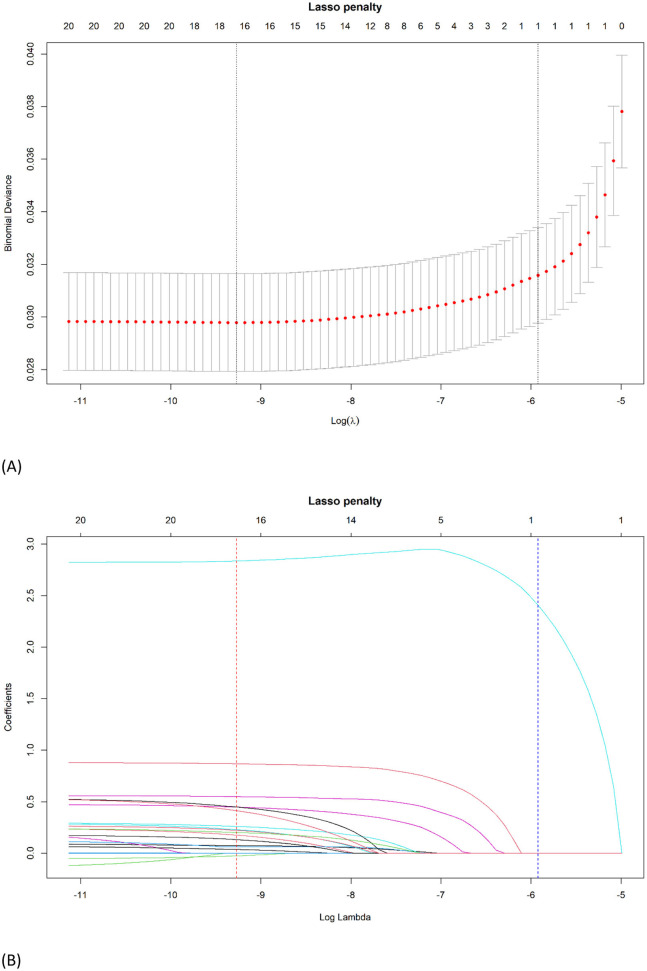
Identification of the optimal penalization coefficient λ in the LASSO logistic regression. **(A)** The LASSO coefficient profiles of the 22 variables. Child's age, sex, race, low birth weight, BMI, having a genetic or inherited condition identified through a blood test, anxiety, asthma, allergy to food, drug, or insect, Type 1 Diabetes, heart condition, household's ability to afford the food you need during the past 12 months, chronic physical pain, difficulty with eating or swallowing in the past 12 months, adequacy of current insurance coverage, and child with a personal doctor or nurse were selected using LASSO binary logistic regression analysis. The LASSO coefficient profiles of the features were plotted. **(B)** The optimum parameter (lambda) selection in the LASSO model performed 10-fold cross-validation through minimum criteria. The partial likelihood deviance (binomial deviance) curve was presented versus log (lambda). Dotted vertical lines were shown at the optimum values by performing the lambda.min (red) and the lambda.1se (blue).

**Figure 2:**
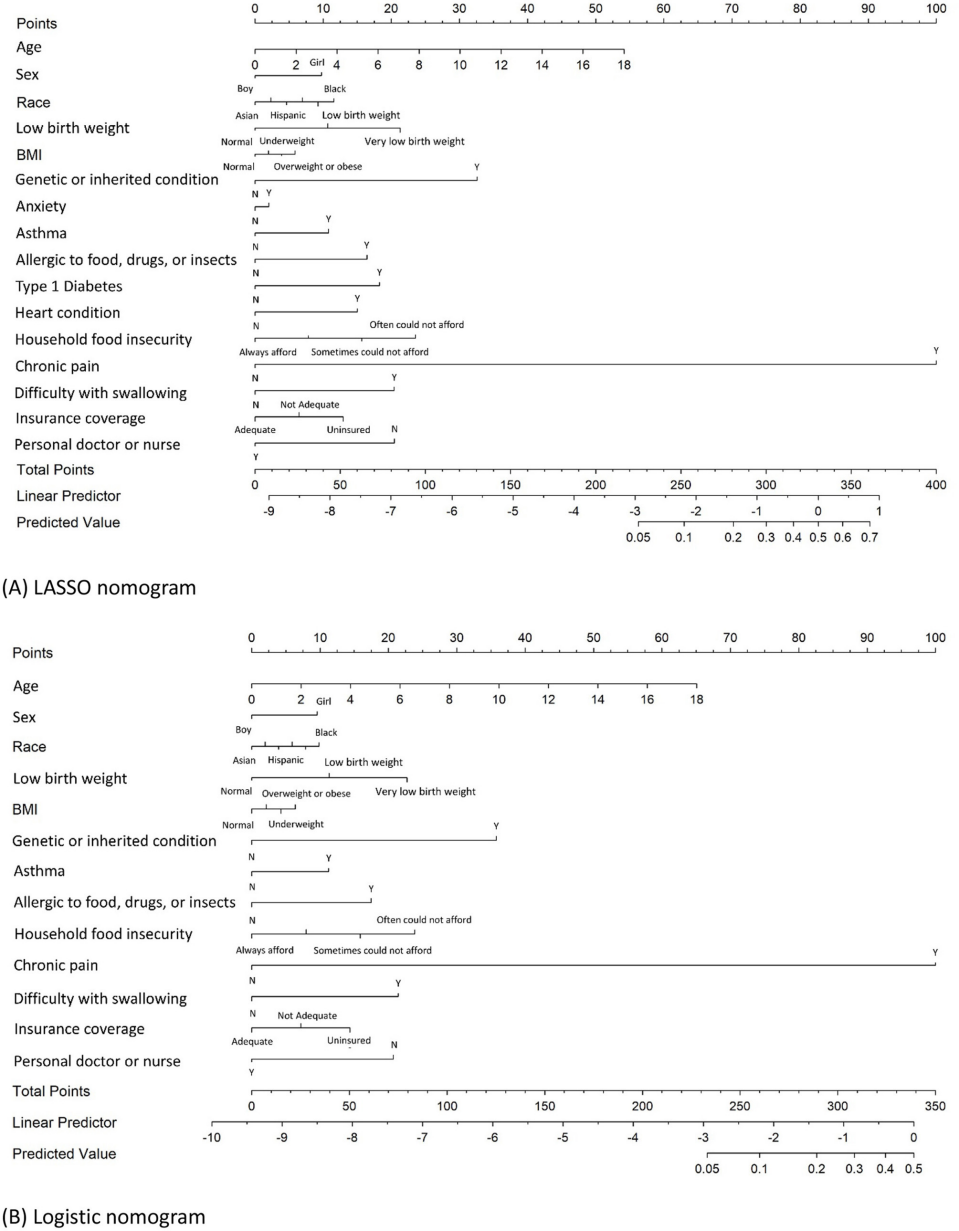
Nomograms for predicting JIA developed by LASSO logistic and logistic regression analysis. **(A)** Nomogram developed by LASSO logistic regression to predict JIA; **(B)** nomogram developed by logistic stepwise regression to predict JIA.

Of the 22 variables included in the LASSO logistic regression analysis, 16 were shown to be statistically significant (p < 0.05) with optimal minimum λ value = 0.0000941 (Figures 1A, B). The JIA predictive nomogram (Figure 2A) was constructed using these 16 variables, i.e., child's age, sex, race, low birth weight, BMI, having a genetic or inherited condition identified through a blood test, anxiety, asthma, allergy to food, drug, or insect, Type 1 Diabetes, heart condition, household's ability to afford the food you need during the past 12 months, chronic physical pain, difficulty with eating or swallowing in the past 12 months, adequacy of current insurance coverage, and child with a personal doctor or nurse. The associated ORs and 95% CIs for each predictor by multivariable logistic regression are demonstrated in Table 3.

**Table 3:** Multivariable logistic regression analysis of JIA predictors identified through LASSO logistic regression and logistic stepwise selection methods.

**Variables**	**LASSO selection method OR (95% CI)**	***p*-value**	**Logistic stepwise selection method OR (95% CI)**	***p*-value**
**Child's age when survey**	1.12 (1.06–1.19)	<0.0001^*^	1.15 (1.09–1.21)	<0.0001^*^
**Sex**				
Boy	1		1	
Girl	1.34 (1.06–1.69)	0.0136^*^	1.33 (1.06–1.67)	0.0140^*^
**Race**				
Asian, non-Hispanic	1		1	
Hispanic	2.06 (0.62–6.85)	0.6483	2.05 (0.62–6.81)	0.5865
White, non-Hispanic	3.14 (1.00–9.86)	0.0500	3.16 (1.01–9.93)	0.0526
Black, non-Hispanic	3.59 (1.07–11.99)	0.0440^*^	3.59 (1.08–11.99)	0.0486^*^
American Indian or Alaska Native Non-Hispanic	3.24 (0.64–16.56)	0.4888	3.28 (0.64–16.77)	0.4926
Others	1.86 (0.53–6.50)	0.4424	1.99 (0.57–6.88)	0.5599
**Low birth weight**				
No	1		1	
Low birth weight	1.45 (1.02–2.06)	0.5484	1.48 (1.04–2.10)	0.5178
Very low birth weight	1.57 (0.79–3.09)	0.4536	1.61 (0.82–3.15)	0.4219
**BMI**				
Normal weight	1		1	
Children age 0–9 years	1.52 (0.89–2.60)	0.4111	1.63 (0.95–2.81)	0.2973
Underweight	1.26 (0.74–2.15)	0.9414	1.27 (0.75–2.15)	0.8469
Overweight or obese	1.41 (1.09–1.83)	0.4252	1.45 (1.12–1.88)	0.4127
**Child's household food insecurity**				
Always afford to eat good nutritious meals	1		1	
Always afford enough to eat but not always the kinds of food we should eat	1.12 (0.87–1.45)	0.1165	1.12 (0.87–1.44)	0.1194
Sometimes could not afford enough to eat	1.62 (1.08–2.44)	0.3212	1.65 (1.10–2.46)	0.2564
Often could not afford enough to eat	1.94 (0.95–3.95)	0.1978	1.87 (0.92–3.79)	0.2383
**Adequacy of current insurance coverage**				
Adequate	1		1	
Not adequate	1.27 (1.01–1.61)	0.4913	1.29 (1.03–1.63)	0.5288
Uninsured	1.30 (0.77–2.21)	0.5928	1.37 (0.82–2.29)	0.4657
**Children with a personal doctor or nurse (Yes vs. No)**	0.58 (0.41–0.82)	0.0019^*^	0.58 (0.41–0.81)	0.0015^*^
**Children's health condition (Yes vs. No)**				
Anxiety	1.03 (0.80–1.33)	0.8203	–	–
Allergy to food, drug, or insect	1.58 (1.21–2.06)	0.0009^*^	1.61 (1.24–2.10)	0.0004^*^
Asthma	1.35 (0.90–2.02)	0.1488	1.36 (0.91–2.04)	0.1321
Chronic physical pain in the past 12 months	16.07 (12.27–21.05)	<0.0001^*^	15.83 (12.15–20.63)	<0.0001^*^
Type 1 Diabetes	1.53 (0.68–3.45)	0.3040	–	–
Difficulty with eating or swallowing in the past 12 months	1.78 (1.22–2.59)	0.0027^*^	1.82 (1.26–2.63)	0.0014^*^
Genetic or inherited condition	2.45 (1.85–3.24)	<0.0001^*^	2.65 (2.02–3.48)	<0.0001^*^
Heart condition	1.52 (0.94–2.46)	0.0892	–	–

* p < 0.05.

OR, Odds Ratio.

CI, Confidence Interval.

### 3.2 Nomogram variables derived from stepwise logistic regression

A stepwise logistic regression analysis was conducted as the second method to identify the significant predictors of JIA. In this analysis, 22 variables were again considered using a stepwise model selection approach with a significant entry and exit level of 0.15. The stepwise logistic regression yielded 13 variables to construct a JIA predictive nomogram. These variables included the child's age, sex, race, low birth weight, BMI, having a genetic or inherited condition identified through a blood test, asthma, allergy to food, drug, or insect, household's ability to afford the food you need during the past 12 months, chronic physical pain, difficulty with eating or swallowing in the past 12 months, adequacy of current insurance coverage, and child with a personal doctor or nurse (Figure 2B). Table 3 demonstrates the ORs for each predictor derived from the multivariable logistic regression.

### 3.3 Prediction of JIA by nomogram

The process of developing the nomogram includes identifying predictor variables located on the relevant axis (e.g., children's age). A straight line is then drawn upward from the value of the result to the Points axis on the top of the nomogram to determine the score received based on the children's age variable. Next, we repeat the above process to all identified predictors, and the total scores are calculated by summing up each predictor's scores. Searching for the total score on the Total points axis. At last, we draw a straight line down from there to obtain the risk of JIA. Using the LASSO nomogram as an example, a 14-year-old (42 points) Black (12 points) girl (10 points). She was born with a very low birth weight (21 points) and was found to have a genetic or inherited condition identified through a blood test at birth (33 points). Her family often could not afford enough to eat during the past 12 months at the survey (24 points). She is allergic to food, drugs, or insects (16 points), has asthma (11 points), has anxiety (2 points), has chronic physical pain (100 points), has Type 1 Diabetes (18 points), has difficulty with eating or swallowing in the past 12 months (20 points), having inadequate insurance coverage (13 points), and having no personal doctor or nurse (20 points). The total score is 342 points, indicating a JIA-predicted probability of 57.9% (Figure 2A).

### 3.4 Prediction of selected model performance

**Figure 3:**
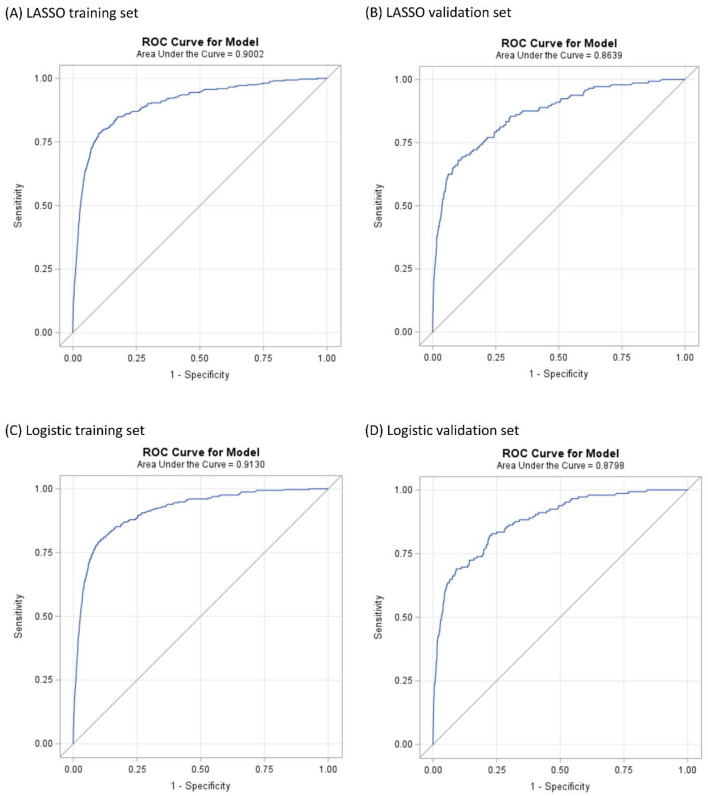
ROC curves illustrating the capability in predicting JIA. **(A, B)** are the result of LASSO logistic regression. **(C, D)** are the result of logistic regression.

In the LASSO logistic regression training set, the ROC curve reveals that the resulting model has excellent discrimination with an area under the curve (AUC) of 0.9002 (95% CI: 0.8814–0.9191) (Figure 3A). The validation set also shows excellent discrimination in LASSO logistic regression, with 0.8639 (95% CI: 0.8310–0.8967) AUC (Figure 3B). The optimal Youden's J cut-off value of this nomogram's sensitivity, specificity, and accuracy were 78.4%, 89.8%, and 89.8% in the training set; and 68.1%, 89.8%, and 89.8% in the validation set. Of the 16 variables selected in the LASSO logistic regression model, 13 were shared with the stepwise logistic regression model (except anxiety, Type 1 Diabetes, and heart condition). The training set of stepwise logistic regression has an AUC of 0.9130 (95% CI: 0.8968–0.9292) (Figure 3C), and the validation set also shows excellent discrimination with 0.8798 (95%CI: 0.8507–0.9088) AUC. The sensitivity, specificity, and accuracy were 79.1%, 90.2%, and 90.2%, respectively, in the training set; and 69.0%, 90.9%, and 90.8% in the validation set (Figure 3D).

**Figure 4:**
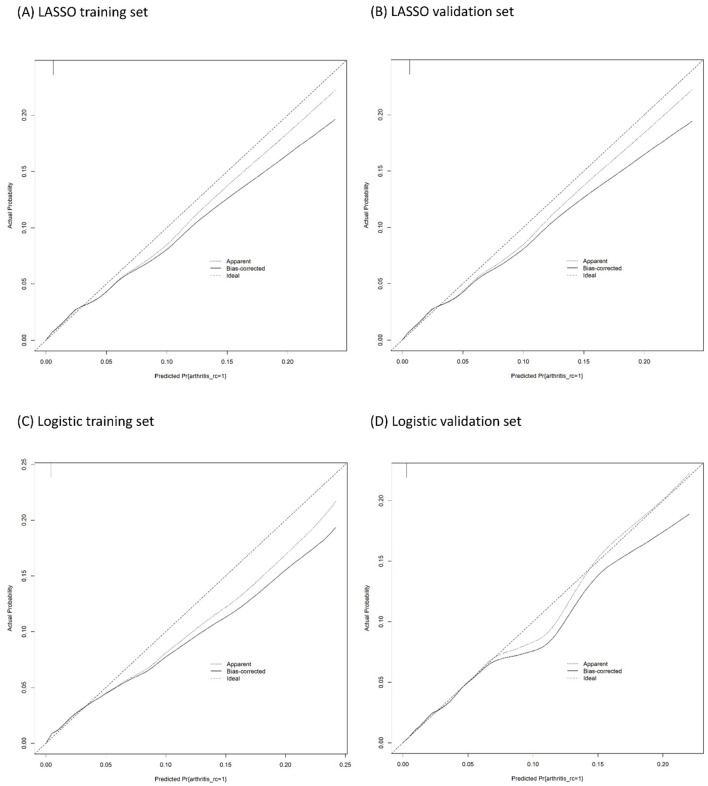
Calibration plots of the binary fringe plot with 1,000 bootstrapping re-sample of LASSO logistic regression for JIA. **(A, B)** are the result of LASSO logistic regression. **(C, D)** are the result of logistic regression. The X-axis showed the predicted probability of JIA. The Y-axis showed the actual probability of JIA. The solid line indicates the performance of the developed nomogram model.

**Figure 5:**
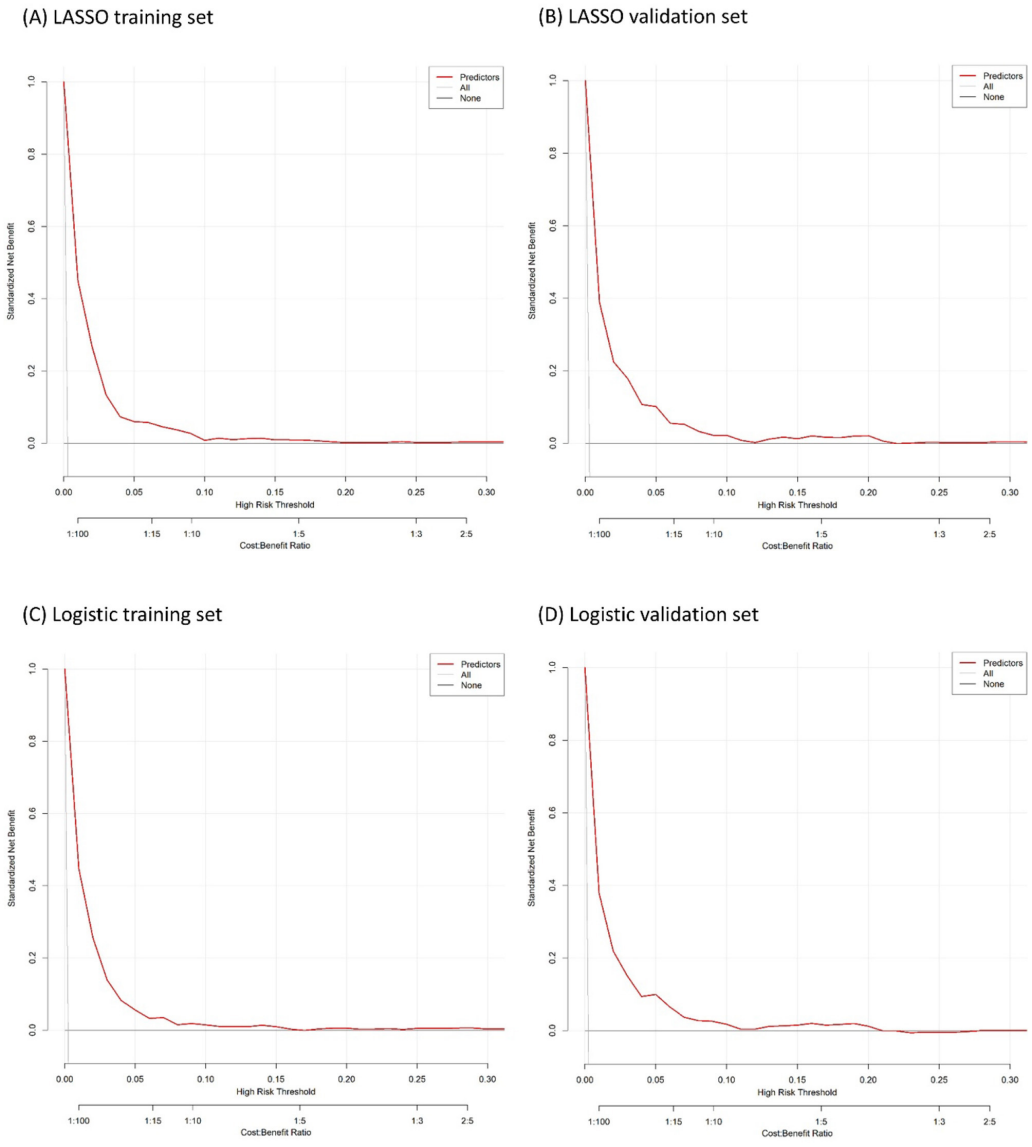
Decision curve analysis for the developed nomogram model. **(A, B)** are the result of LASSO logistic regression. **(C, D)** are the result of logistic regression.

The calibration plots of models were used to provide better information about the selected models, graphically showing good agreement between the predicted and observed data in the training and validation cohorts (Figure 4). Furthermore, the Decision Curve Analyses (DCA) present significant net benefits of the predictive LASSO and stepwise logistic models in the training set (Figures 5A, C) and validation set (Figures 5B, D). These findings demonstrated that our nomograms had significant potential for clinical use.

## 4 Discussion

In this study, the authors utilized the National Survey of Children's Health (NSCH) database, which included 223,195 children across all 50 states in the US from January 1, 2016, to December 31, 2021. The aim was to develop a predictive nomogram for juvenile idiopathic arthritis (JIA) using LASSO and stepwise logistic regression methods. The LASSO and stepwise logistic regression models identified several independent predictors for JIA, including age, sex, race/ethnicity, low birth weight, BMI, genetic or inherited conditions, asthma, food, drug, or insect allergies, household food insufficiency, chronic physical pain, difficulty swallowing or eating, and inadequate insurance coverage. Having a personal doctor or nurse was a protective factor for JIA. The LASSO logistic regression included anxiety, Type 1 Diabetes, and heart condition as predictors for JIA, which the stepwise logistic regression modeling did not choose, suggesting that these three variables were at the borderline of significance.

Stepwise logistic regression, integrating both forward selection and backward elimination, is a commonly used method for variable selection in public health, medicine, economics, and social sciences research (52), particularly in diagnostic studies. This approach aims to balance model simplicity and predictive accuracy by iteratively adding and removing variables based on statistical significance and information criteria. It requires less computational power and time than complex machine learning models, making it a practical choice for large datasets. However, studies have found that a larger number of predicting variables undermine its effectiveness and lead to unstable parameter estimates (50, 53, 54). To this end, alternative methods like penalized regression models (e.g., LASSO) have been suggested, offering more robust and efficient variable selection (54). The LASSO logistic regression is a statistical technique that has gained popularity due to its effectiveness in tackling the overfitting issue in regression analysis when there are many predictors or when some variables are highly correlated. By introducing a penalty parameter to the usual likelihood function, the LASSO method can effectively shrink the coefficients of less important variables to zero, resulting in a more parsimonious model. This approach also helps avoid overestimating the model's performance and reduces the complexity of high-dimensional data, making it easier to interpret and understand (44). Several studies have used stepwise logistic regressions to build nomograms (55–59) and compared them to the LASSO logistic regression results (60). The authors concluded that both LASSO and stepwise logistic regression are suitable for selecting and comparing predictors to screen potential children with JIA early.

This study found that both age and sex were predictors for JIA. We discovered that age was positively associated with the probability of being diagnosed with JIA, which is consistent with the previous study (39). JIA rarely occurs in babies younger than 6 months (61–63). The first peak of JIA is between the ages of 2 and 5 years, and the second is between 6 and 14 years (13, 64–66). Although the term juvenile in JIA refers to the fact that it typically begins in childhood, the condition can continue into adolescence and adulthood for specific individuals (67). Several studies have evidenced that sex is a risk factor for JIA (16–18, 68), especially in the oligoarticular and rheumatoid factor (RF) negative polyarticular subtypes (69). Oligoarticular is the most common JIA subtype in developed countries, typically affecting girls under 6 years old (30). The RF-negative polyarticular JIA shows a bimodal trend in girls (64). Sex differences in age peaks were also observed. Some studies indicated no peaks for any age group at diagnosis for boys (4), but for girls, two small peaks appeared at ages zero to 5 years and 12–15 years (39).

The LASSO and logistic models selected predictive factors for JIA, such as low birth weight, BMI, food affordability in the past 12 months, and difficulty with eating or swallowing in the past year. In previous studies, these factors were also associated with JIA. Low birth weight, defined as weight < 2,500 grams, has been linked to a higher risk of developing JIA (70–74). On the other hand, different studies found that high birth weight was also associated with a significantly decreased risk of JIA overall (73, 74). However, what causes such a difference in low and high birth weights (73) is unclear. In this study, we did not have information regarding high birth weight. We discovered that children with very low birth weight had a higher risk of JIA than those with low birth weight. Obesity was reported to be increased in children and young people with JIA (75). Children with physical disabilities may have an increased risk for obesity, which in turn might be a risk factor for inflammatory arthritis in obese children compared to normal-weight children (76, 77). A possible relationship between adipose tissue and inflammatory arthritis is through the role of adipokines (76). We also found that being underweight is associated with JIA. This finding is similar to the observation of other studies indicating that children with arthritis had significantly lower weight and height than healthy controls (75).

Lack of proper nutrition can affect the immune system, potentially influencing the development of autoimmune conditions (78, 79). Household food insufficiency is when a family does not have enough food to meet their nutritional needs (80). This insufficiency is an environmental factor that can increase the risk of JIA, as it can be related to malnutrition, which negatively affects the immune system, joints, and growth (78, 79). Research has indicated that food insecurity correlates with elevated levels of proinflammatory cytokines, suggesting a possible pathway through which food insecurity could influence the onset or worsening of autoimmune diseases (81). Furthermore, malnutrition, especially vitamin D deficiency, has been linked to the development of autoimmune diseases. Vitamin D is essential for immune modulation, and its deficiency has been associated with a higher risk of autoimmune disorders, such as JIA. Studies have shown that low levels of vitamin D can affect immune cell activity, potentially contributing to the emergence of autoimmune diseases (82). Difficulty swallowing or eating (dysphagia) is when someone has trouble moving food or liquids from the mouth to the stomach. Dysphagia can lead to mechanical feeding difficulties, such as trouble with chewing and swallowing, which narrows food choices and impairs nutritional intake, further exacerbating disease symptoms. Chronic inflammation in these diseases can cause damage to organs, including the gastrointestinal tract, through both the direct action of autoantibodies and the side effects of pharmacological therapies (75, 83, 84).

Things may get worse when a child has asthma, a risk factor for JIA, as it can trigger or worsen the immune response and inflammation in the joints. Some autoimmune diseases might share common genetic or environmental triggers with allergic conditions (85–87). Children with JIA were found to have increased levels of activated CD4+ T-cells both in circulation and synovium (88–91). Allergies can also increase the risk of JIA, as they can activate the immune system and cause joint inflammation (87). Both asthma and allergy were selected by LASSO logistic regression to predict JIA in our study.

We found that anxiety is associated with JIA. Our finding was consistent with previous studies (92). The prevalence of symptoms of stress in youth with JIA ranged from 7% to 64% (93). JIA patients have been found to have increased enzyme activity in dopamine and serotonin metabolism, which may explain a tendency to be associated with depression, anxiety disorders, and cognitive impairment (92, 94). Immune deficiencies can also have an impact on blood flow to the brain. Patients with immune deficiencies experience a significant decrease in the size of the right frontal and right parietal lobes.

In contrast, the size of the left parietal and occipital lobes increases significantly compared to the control group. These regions of the brain are known to be involved in anxiety (95). In addition, the long duration of illness is found to be associated with a higher proportion of psychiatric disorders (95). Although previous studies connected depression and JIA (92–95), we did not find depression was associated with JIA in this present study.

The LASSO logistic regression also included Type 1 Diabetes Mellitus (T1D) and heart condition as predictors of JIA. JIA and T1D are both autoimmune diseases and can coexist in the same individual. Studies have reported a higher prevalence of T1D in patients with JIA compared to non-JIA groups (96). Modern diabetes technologies like personal insulin pumps and continuous glucose monitoring can help to minimize the deteriorating effect of JIA exacerbations and rheumatoid treatment on metabolic control of diabetes (97). JIA has been found to be associated with cardiovascular disorders (98–100), which is consistent with our findings that having a heart condition is a predictive factor for JIA. Heart conditions can increase the risk of JIA by causing inflammation, infection, and stress that can affect the immune system and the joints (14, 99). Patients with JIA are known to experience pericardial, myocardial, or endocardial involvements (14, 98). Endocarditis, for instance, can cause aortic regurgitation or mitral regurgitation, which may require valve surgery (101, 102). Furthermore, recent studies show that young patients with rheumatologic disorders have become more susceptible to ischemic coronary artery diseases due to premature atherosclerosis (98).

Both LASSO and stepwise logistic regression selected chronic pain to predict JIA in our study. Pain in JIA is multifactorial. Chronic pain in pediatrics is commonly defined as prolonged pain that lasts longer than 3 months or any recurrent pain that occurs at least three times throughout 3 months. A study found that 39% of patients reported pain on all diary days over the 8 weeks, while only 5 % reported no pain over the study period (103, 104). Children with persistent pain due to JIA experience significantly more problems with physical, emotional, social, and school functioning than healthy individuals (32). JIA itself causes joint pain and inflammation. However, having chronic pain from other sources might exacerbate the overall discomfort experienced by a person with JIA (101, 102). Chronic physical pain can increase the risk of JIA, as it can alter the pain perception and the nervous system, influencing inflammation and joint function (103, 104).

The financial cost of JIA can be high (10). Inadequate insurance coverage can limit access to healthcare, diagnosis, treatment, and follow-up, worsening disease outcomes and complications in JIA patients. There are discrepancies in healthcare access, as people with public insurance have worse results, such as a greater chance of long-term functional disability (105). Despite extensive research, there is no single definitive test that doctors can use to diagnose JIA. However, physicians may suspect that a child has the disease during multiple visits if they present with unexplained joint pain, stiffness, or swelling that has persisted for at least six weeks. The diagnosis of JIA is typically based on a combination of clinical evaluation, laboratory tests, and imaging studies (25, 30). It is highly beneficial for children with JIA to visit their primary care physicians or nurses whenever needed, as early diagnosis and ongoing medical care can help control their condition to prevent joint damage and improve long-term outcomes (30, 106). Numerous studies have also shown that detecting and treating JIA early can alleviate joint pain and stiffness, enabling children to move and function more comfortably. This, in turn, can improve their overall wellbeing and ability to participate in everyday activities. Early treatment can also lead to better long-term outcomes for children with JIA, reducing the risk of complications and the need for surgical interventions such as joint replacements (10, 107). Timely diagnosis and treatment can also help reduce overall healthcare costs associated with JIA, as effective management can lead to fewer hospitalizations, surgeries, and other expensive interventions (29). JIA can have an impact on a child's growth and development, particularly in the case of sizeable joint involvement. However, early treatment can mitigate these effects and support normal growth patterns. While various diseases, such as asthma, allergies, diabetes, and heart disease, have been found to be associated with JIA, this study did not explore in depth how these comorbidities interact to influence the risk of JIA. Future research should further investigate these interactions.

### 4.1 Study limitation

This study has several limitations: (1) It is a repeated cross-sectional study focusing on predictive modeling to understand associations between measured factors, and therefore, it does not evaluate causal pathways or make causal inferences. Potential causal associations, such as the associations of JIA with obesity, inflammation, anxiety, asthma, allergy, heart condition, chronic pain, and household food insufficiency, require additional studies to establish; (2) the questions in the survey questionnaire to identify the diagnosis of arthritis did not mentioned the term “idiopathic.” Also, parents' familiarity with arthritis was not measured in the survey. These could be possible sources of information bias. (3) Approximately 1% (n = 2,248) of children in the NSCH dataset were excluded from the current analysis due to missing responses to JIA-related questions from their parents. Excluding this missing data could introduce selection bias, potentially stemming from a collider effect. However, further investigation is required to determine whether the missingness was associated with the predictor variables and the outcome. To assess potential bias, we compared children with and without missing JIA information in the data based on the predictor variables selected by LASSO logistic regression (Supplementary Table 2). While some variables indicated significant differences between the non-missing and missing groups, the authors still could not conclusively establish the presence of selection bias as we lacked information regarding the association between JIA and the collider (reason of missing). On the other hand, the NSCH weighting adjustments were generally effective in minimizing the nonresponse bias and enhancing the survey's representativeness (34, 38–40). (4) This dataset did not include genetic and some perinatal information. JIA is a complex genetic disease that does not demonstrate a single gene-based Mendelian inheritance pattern (13). Extensive review has been conducted on genetic variants, such as PTPN22, HLA-A2, HLA-B27, HLA DRB1^*^01, DRB1^*^08, DRB1^*^11, DRB1^*^13, DPB1^*^02, and DQB1^*^04, that contribute to JIA susceptibility (1, 108, 109). This information was not collected in the NSCH; (5) this study did not have information about the JIA subtypes. Each subtype can have varying predictive factors, characteristics, and degrees of severity and may require different treatment approaches; (6) due to still existing trends in the under-diagnosis of JIA, this study may have missed some undiagnosed children with JIA in the comparison group. This can underestimate our findings; (7) the nomograms were developed based on data from U.S. children. Therefore, caution should be exercised when applying these tools to children outside the U.S., as differences in demographics, healthcare systems, and other factors may affect their generalizability.

## 5 Conclusion

Using two well-validated predictor models, we developed nomograms for the early prediction of JIA in children based on NSCH database data. The tools are also available for parents and health professionals to utilize these nomograms. Our easy-to-use nomograms are not intended to replace the standard diagnostic methods. Still, they are designed to assist parents, clinicians, and researchers in better-estimating children's potential risk of JIA. We advise individuals utilizing our nomogram model to be mindful of potential pre-existing selection biases that may affect referrals and diagnoses.

## Data Availability

The datasets presented in this study can be found in online repositories. The names of the repository/repositories and accession number(s) can be found below: The anonymized NSCH data collected are available as open databases via https://www.childhealthdata.org/dataset/download?rq=16239.

## References

[1] Zhang W Cai Z Liang D Han J Wu P Shan J. Immune cell-related genes in juvenile idiopathic arthritis identified using transcriptomic and single-cell sequencing data. Int J Mol Sci. (2023) 24:10619. 10.3390/ijms241310619 37445800 PMC10342059

[2] Martini A Lovell DJ Albani S Brunner HI Hyrich KL Thompson SD. Juvenile idiopathic arthritis. Nat Rev Dis Primers. (2022) 8:5. 10.1038/s41572-021-00332-8 35087087

[3] Thatayatikom A De Leucio A. Juvenile Idiopathic Arthritis (JIA). Bethesda, MD: StatPearls, National Library of Medicine (NLM). (2020).

[4] Berntson L Gäre BA Fasth A Herlin T Kristinsson J Lahdenne P. Incidence of juvenile idiopathic arthritis in the Nordic countries. A population based study with special reference to the validity of the ILAR and EULAR criteria. J Rheumatol. (2003) 30:2275–82. 14528529

[5] Danner S Sordet C Terzic J Donato L Velten M Fischbach M. Epidemiology of juvenile idiopathic arthritis in Alsace, France. J Rheumatol. (2006) 33:1377–81. 16821272

[6] Pruunsild C Uibo K Liivamägi H Tarraste S Talvik T Pelkonen P. Incidence of juvenile idiopathic arthritis in children in Estonia: a prospective population-based study. Scand J Rheumatol. (2007) 36:7–13. 10.1080/03009740601089259 17454929

[7] Hanova P Pavelka K Dostal C Holcatova I Pikhart H. Epidemiology of rheumatoid arthritis, juvenile idiopathic arthritis and gout in two regions of the Czech Republic in a descriptive population-based survey in 2002–2003. Clin Exp Rheumatol. (2006) 24:499–507. 17181917

[8] Cimaz R. Systemic-onset juvenile idiopathic arthritis. Autoimmun Rev. (2016) 15:931–4. 10.1016/j.autrev.2016.07.004 27392503

[9] Lites TD. Arthritis among children and adolescents aged 18 years—United States, 2017–2021. MMWR Morb Mortal Wkly Rep. (2023) 72:788–92. 10.15585/mmwr.mm7229a3 37471260 PMC10360652

[10] García-Rodríguez F Gamboa-Alonso A Jiménez-Hernández S Ochoa-Alderete L Barrientos-Martínez VA Alvarez-Villalobos NA. Economic impact of Juvenile Idiopathic Arthritis: a systematic review. Pediatric Rheumatol. (2021) 19:1–10. 10.1186/s12969-021-00641-y 34627296 PMC8502332

[11] Petty RE Southwood TR Manners P Baum J Glass DN Goldenberg J. International League of Associations for Rheumatology classification of juvenile idiopathic arthritis: second revision, Edmonton, 2001. J Rheumatol. (2004) 31:390–2. 14760812

[12] Malattia C Rinaldi M Martini A. The role of imaging in juvenile idiopathic arthritis. Expert Rev Clin Immunol. (2018) 14:681–94. 10.1080/1744666X.2018.1496019 29972659

[13] Ellis JA Munro JE Ponsonby A-L. Possible environmental determinants of juvenile idiopathic arthritis. Rheumatology. (2010) 49:411–25. 10.1093/rheumatology/kep383 19965974

[14] Zaripova LN Midgley A Christmas SE Beresford MW Baildam EM Oldershaw RA. Juvenile idiopathic arthritis: from aetiopathogenesis to therapeutic approaches. Pediat Rheumatol. (2021) 19:1–14. 10.1186/s12969-021-00629-8 34425842 PMC8383464

[15] Clarke SL Sen ES Ramanan AV. Juvenile idiopathic arthritis-associated uveitis. Pediat Rheumatol. (2016) 14:1–11. 10.1186/s12969-016-0088-2 27121190 PMC4848803

[16] Clinic M. Juvenile Idiopathic Arthritis. Available online at: https://www.mayoclinic.org/diseases-conditions/juvenile-idiopathic-arthritis/symptoms-causes/syc-20374082 (accessed August 20, 2024).

[17] Cattalini M Soliani M Caparello MC Cimaz R. Sex differences in pediatric rheumatology. Clin Rev Allergy Immunol. (2019) 56:293–307. 10.1007/s12016-017-8642-3 28849549

[18] Al-Mayouf SM Al Mutairi M Bouayed K Habjoka S Hadef D Lotfy HM. Epidemiology and demographics of juvenile idiopathic arthritis in Africa and Middle East. Pediat Rheumatol. (2021) 19:1–30. 10.1186/s12969-021-00650-x 34857004 PMC8638433

[19] Sen ES Dick AD Ramanan AV. Uveitis associated with juvenile idiopathic arthritis. Nat Rev Rheumatol. (2015) 11:338–48. 10.1038/nrrheum.2015.20 25825278

[20] Ayuso VK Makhotkina N van Tent-Hoeve M de Groot-Mijnes JD Wulffraat NM Rothova A. Pathogenesis of juvenile idiopathic arthritis associated uveitis: the known and unknown. Surv Ophthalmol. (2014) 59:517–31. 10.1016/j.survophthal.2014.03.002 25130893

[21] Long AM Marston B. Juvenile idiopathic arthritis. Pediat Rev. (2023) 44:565–77. 10.1542/pir.2022-005623 37777651

[22] Kimura Y. Systemic Juvenile Idiopathic Artrhritis: Clinical Manifestations and Diagnosis. (2017). Waltham, MA: UpToDate.

[23] Dimitriou C Boitsios G Badot V Lê P-Q Goffin L Simoni P. Imaging of juvenile idiopathic arthritis. Radiol Clinics. (2017) 55:1071–83. 10.1016/j.rcl.2017.04.011 28774449

[24] Mehta J. Juvenile Idiopathic Arthritis. Available online at: https://www.msdmanuals.com/professional/pediatrics/juvenile-idiopathic-arthritis/juvenile-idiopathic-arthritis-jia (accessed August 17, 2024).

[25] National Institute of Arthritis and Musculoskeletal and Skin Diseases. Juvenile Idiopathic Arthritis (JIA): Diagnosis, Treatment, and Steps to Take. Available online at: https://www.niams.nih.gov/health-topics/juvenile-arthritis/diagnosis-treatment-and-steps-to-take (accessed August 17, 2024).

[26] Foster H Kay L May C Rapley T. Pediatric regional examination of the musculoskeletal system: a practice-and consensus-based approach. Arthritis Care Res. (2011) 63:1503–10. 10.1002/acr.20569 21954040

[27] Garner AJ Saatchi R Ward O Hawley DP. Juvenile idiopathic arthritis: a review of novel diagnostic and monitoring technologies. Healthcare. (2021) 9:1683. 10.3390/healthcare9121683 34946409 PMC8700900

[28] Aoust L Rossi-Semerano L Koné-Paut I Dusser P. Time to diagnosis in juvenile idiopathic arthritis: a French perspective. Orphanet J Rare Dis. (2017) 12:1–5. 10.1186/s13023-017-0586-4 28241879 PMC5329952

[29] Ong M-S Schiff G Natter M. Incidence, contributing factors, and impact of diagnostic delay in juvenile idiopathic arthritis: analysis of the Childhood Arthritis and Rheumatology Research Alliance (CARRA) registry. In: Arthritis & Rheumatology. Hoboken NJ: Wiley. (2020).

[30] Giancane G Consolaro A Lanni S Davi S Schiappapietra B Ravelli A. Juvenile idiopathic arthritis: diagnosis and treatment. Rheumatol Therapy. (2016) 3:187–207. 10.1007/s40744-016-0040-4 27747582 PMC5127964

[31] Prakken B Albani S Martini A. Juvenile idiopathic arthritis. Lancet. (2011) 377:2138–49. 10.1016/S0140-6736(11)60244-4 21684384

[32] Weiss JE Luca NJ Boneparth A Stinson J. Assessment and management of pain in juvenile idiopathic arthritis. Pediatric Drugs. (2014) 16:473–81. 10.1007/s40272-014-0094-0 25331986

[33] Li X-D Li M-M A. novel nomogram to predict mortality in patients with stroke: a survival analysis based on the MIMIC-III clinical database. BMC Med Inform Decis Mak. (2022) 22:92. 10.1186/s12911-022-01836-3 35387672 PMC8988376

[34] NSCH. National Survey of Children's Health. (2016). Available online at: https://www.childhealthdata.org/learn-about-the-nsch/methods (accessed August 7, 2024).

[35] Gurney JG McPheeters ML Davis MM. Parental report of health conditions and health care use among children with and without autism: National Survey of Children's Health. Arch Pediatr Adolesc Med. (2006) 160:825–30. 10.1001/archpedi.160.8.825 16894082

[36] National Survey of Children's Health Interactive Data Query. NSCH Survey Methodology. (2024). Available online at: https://www.childhealthdata.org/learn-about-the-nsch/methods (accessed August 7, 2024).

[37] The United States Census Bureau ADoDP, National Survey of Children's Health. National Survey of Children's Health Frequently Asked Questions. (2022). Available online at: https://www.census.gov/programs-surveys/nsch/data/datasets.html (accessed August 8, 2024).

[38] Martini A Ravelli A Avcin T Beresford MW Burgos-Vargas R Cuttica R. Toward new classification criteria for juvenile idiopathic arthritis: first steps, pediatric rheumatology international trials organization international consensus. J Rheumatol. (2019) 46:190–7. 10.3899/jrheum.180168 30275259

[39] Cardoso I Frederiksen P Specht IO Händel MN Thorsteinsdottir F Heitmann BL. Age and sex specific trends in incidence of juvenile idiopathic arthritis in Danish birth cohorts from 1992 to 2002: a nationwide register linkage study. Int J Environ Res Public Health. (2021) 18:8331. 10.3390/ijerph18168331 34444082 PMC8394352

[40] Bureau UC. 2020 National Survey of Children's Health: Nonresponse Bias Analysis. Washington, DC: US Department of Commerce, Economics and Statistics Administration. (2021).

[41] Wang X Cheng Z. Cross-sectional studies: strengths, weaknesses, and recommendations. Chest. (2020) 158:S65–71. 10.1016/j.chest.2020.03.012 32658654

[42] Pan X. Repeated cross-sectional design. In: Encyclopedia of Gerontology and Population Aging. Cham: Springer (2022). p. 4246–50. 10.1007/978-3-030-22009-9_578

[43] Yee JL Niemeier D. Advantages and Disadvantages: Longitudinal vs. Repeated Cross-Section Surveys. Washington, DC: United States Federal Highway Administration (1996).

[44] Stock JH Watson MW. Introduction to Econometrics. London: Pearson (2020).

[45] Breiman L. Classification and Regression Trees. London: Routledge (2017).

[46] Lai L Su T Liang Z Lu Y Hou E Lian Z. Development and assessment of novel predictive nomograms based on APRI for hepatitis B virus-associated small solitary hepatocellular carcinoma with stereotactic body radiotherapy. J Cancer. (2020) 11:6642. 10.7150/jca.47291 33046985 PMC7545675

[47] Wang Q Qiao W Zhang H Liu B Li J Zang C. Nomogram established on account of Lasso-Cox regression for predicting recurrence in patients with early-stage hepatocellular carcinoma. Front Immunol. (2022) 13:1019638. 10.3389/fimmu.2022.1019638 36505501 PMC9726717

[48] Yao R Zheng B Hu X Ma B Zheng J Yao K. Development of a predictive nomogram for in-hospital death risk in multimorbid patients with hepatocellular carcinoma undergoing Palliative Locoregional Therapy. Sci Rep. (2024) 14:13938. 10.1038/s41598-024-64457-y 38886455 PMC11183254

[49] Mao S Yu X Yang Y Shan Y Mugaanyi J Wu S. Preoperative nomogram for microvascular invasion prediction based on clinical database in hepatocellular carcinoma. Sci Rep. (2021) 11:13999. 10.1038/s41598-021-93528-7 34234239 PMC8263707

[50] Wolf BJ Jiang Y Wilson SH Oates JC. Variable selection methods for identifying predictor interactions in data with repeatedly measured binary outcomes. J Clini Transl Sci. (2021) 5:e59. 10.1017/cts.2020.556 33948279 PMC8057419

[51] Mundry R Nunn CL. Stepwise model fitting and statistical inference: turning noise into signal pollution. Am Nat. (2009) 173:119–23. 10.1086/593303 19049440

[52] Butcher B Smith BJ. Feature Engineering and Selection: A Practical Approach for Predictive Models. Boca Raton, FL: Chapman & Hall/CRC Press (2019).

[53] Gary S. Step away from stepwise. J Big Data. (2018) 5:1–12. 10.1186/s40537-018-0143-6

[54] Zabor EC Reddy CA Tendulkar RD Patil S. Logistic regression in clinical studies. Int J Radiat Oncol Biol Phys. (2022) 112:271–7. 10.1016/j.ijrobp.2021.08.007 34416341

[55] Ing EB Ing R. The use of a nomogram to visually interpret a logistic regression prediction model for giant cell arteritis. Neuro-Ophthalmology. (2018) 42:284–6. 10.1080/01658107.2018.1425728 30258473 PMC6152514

[56] Zlotnik A Abraira V A. general-purpose nomogram generator for predictive logistic regression models. Stata J. (2015) 15:537–46. 10.1177/1536867X1501500212

[57] Chun FK-H Graefen M Briganti A Gallina A Hopp J Kattan MW. Initial biopsy outcome prediction—head-to-head comparison of a logistic regression-based nomogram versus artificial neural network. Eur Urol. (2007) 51:1236–43. 10.1016/j.eururo.2006.07.021 16945477

[58] Bertens LC Moons KG Rutten FH van Mourik Y Hoes AW Reitsma JB. nomogram was developed to enhance the use of multinomial logistic regression modeling in diagnostic research. J Clin Epidemiol. (2016) 71:51–7. 10.1016/j.jclinepi.2015.10.016 26577433

[59] Shin M-S Lee J-Y. Building a nomogram for metabolic syndrome using logistic regression with a complex sample—a study with 39,991,680 cases. Healthcare. (2022) 10:372. 10.3390/healthcare10020372 35206986 PMC8871838

[60] Wang X-Y Lin J-J Lu M-K Jang F-L Tseng H-H Chen P-S. Development and validation of a web-based prediction tool on minor physical anomalies for schizophrenia. Schizophrenia. (2022) 8:4. 10.1038/s41537-021-00198-5 35210439 PMC8873231

[61] Roemer J Klein A Horneff G. Prevalence and risk factors of depressive symptoms in children and adolescents with juvenile idiopathic arthritis. Rheumatol Int. (2023) 2023:1–9. 10.1007/s00296-023-05323-4 37039854 PMC10261240

[62] Team AsMK. Juvenile Idiopathic Arthritis. (2022). Available online at: https://ada.com/conditions/juvenile-idiopathic-arthritis/ (accessed September 19, 2024).

[63] Harrold LR Salman C Shoor S Curtis JR Asgari MM Gelfand JM. Incidence and prevalence of juvenile idiopathic arthritis among children in a managed care population, 1996–2009. J Rheumatol. (2013) 40:1218–25. 10.3899/jrheum.120661 23588938 PMC5657479

[64] Ravelli A Martini A. Juvenile idiopathic arthritis. Lancet. (2007) 369:767–78. 10.1016/S0140-6736(07)60363-8 17336654

[65] Sullivan DB Cassidy JT Petty RE. Pathogenic implications of age of onset in juvenile rheumatoid arthritis. Arthritis Rheumat. (1975) 18:251–5. 10.1002/art.1780180309 1137612

[66] Jia P. Polyarticular Juvenile Idiopathic Arthritis: Clinical Manifestations, Diagnosis, and Complications. Waltham, MA: Wolters Kluwer Health (2017).

[67] d'Angelo DM Di Donato G Breda L Chiarelli F. Growth and puberty in children with juvenile idiopathic arthritis. Pediat Rheumatol. (2021) 19:1–13. 10.1186/s12969-021-00521-5 33712046 PMC7953722

[68] Tordoff M Smith S Morris A Eyre S Thomson W Bowes J. OP0193 Sex Dimorphism Analysis in a Cohort Of Jia Patients Reveals Differing Genetic Risk Factors for Females and Males. London: BMJ Publishing Group Ltd. (2023). 7

[69] Barut K Adrovic A Sahin S Kasapçopur Ö. Juvenile idiopathic arthritis. Balkan Med J. (2017) 34:90–101. 10.4274/balkanmedj.2017.0111 28418334 PMC5394305

[70] Uyamasi K Wang K Johnson KR. Family Size and Risk of Juvenile Idiopathic Arthritis: A Cross-Sectional Study. Johnson City, TN: East Tennessee State University (2019).

[71] Carlens C Jacobsson L Brandt L Cnattingius S Stephansson O Askling J. Perinatal characteristics, early life infections and later risk of rheumatoid arthritis and juvenile idiopathic arthritis. Ann Rheum Dis. (2009) 68:1159–64. 10.1136/ard.2008.089342 18957482

[72] Shenoi S Shaffer M Wallace C. Environmental risk factors and early-life exposures in juvenile idiopathic arthritis: a case–control study. Arthritis Care Res. (2016) 68:1186–94. 10.1002/acr.22806 26618899 PMC5515549

[73] Bell SW Shenoi S Nelson JL Bhatti P Mueller BA. Juvenile idiopathic arthritis in relation to perinatal and maternal characteristics: a case control study. Pediatric Rheumatol. (2017) 15:1–7. 10.1186/s12969-017-0167-z 28494794 PMC5425970

[74] Clarke SL Mageean KS Maccora I Harrison S Simonini G Sharp GC. Moving from nature to nurture: a systematic review and meta-analysis of environmental factors associated with juvenile idiopathic arthritis. Rheumatology. (2022) 61:514–30. 10.1093/rheumatology/keab627 34382060 PMC8824412

[75] Zare N Mansoubi M Coe S Najafi AA Bailey K Harrison K. An investigation into the relationship between dietary intake, symptoms and health-related quality of life in children and young people with juvenile idiopathic arthritis: A systematic review and meta-analysis. BMC Pediatr. (2023) 23:3. 10.1186/s12887-022-03810-4 36593466 PMC9806873

[76] Derdemezis C Voulgari P Drosos A Kiortsis D. Obesity, adipose tissue and rheumatoid arthritis: coincidence or more complex relationship. Clin Exp Rheumatol. (2011) 29:712–27. 21640051

[77] Pelajo CF Lopez-Benitez JM Miller LC. Obesity and disease activity in juvenile idiopathic arthritis. Pediat Rheumatol. (2012) 10:1–5. 10.1186/1546-0096-10-3 22240096 PMC3283518

[78] Zandonadi RP. An overview of nutritional aspects in juvenile idiopathic arthritis. Nutrients. (2022) 14:4412. 10.3390/nu14204412 36297096 PMC9610591

[79] Onel KB Horton DB Lovell DJ Shenoi S Cuello CA Angeles-Han ST. 2021 American College of Rheumatology guideline for the treatment of juvenile idiopathic arthritis: therapeutic approaches for oligoarthritis, temporomandibular joint arthritis, and systemic juvenile idiopathic arthritis. Arthritis Rheumatol. (2022) 74:553–69. 10.1002/art.42037 35233993 PMC10161784

[80] Lee SE Song YJ Kim Y Choe J Paik H-Y. Household food insufficiency is associated with dietary intake in Korean adults. Public Health Nutr. (2016) 19:1112–21. 10.1017/S1368980015002438 26299577 PMC10270916

[81] Leddy AM Roque A Sheira LA Frongillo EA Landay AL Adedimeji AA. Food insecurity is associated with inflammation among women living with HIV. J Infect Dis. (2019) 219:429–36. 10.1093/infdis/jiy511 30165648 PMC6325349

[82] Sîrbe C Rednic S Grama A Pop TL. An update on the effects of vitamin D on the immune system and autoimmune diseases. Int J Mol Sci. (2022) 23:9784. 10.3390/ijms23179784 36077185 PMC9456003

[83] Eyigör S. Dysphagia in rheumatological disorders. World J Rheumatol. (2013) 3:45–50. 10.5499/wjr.v3.i3.45

[84] Di Piazza A Vernuccio F Costanzo M Scopelliti L Picone D Midiri F. The videofluorographic swallowing study in rheumatologic diseases: a comprehensive review. Gastroenterol Res Pract. (2017) 2017:7659273. 10.1155/2017/7659273 28706536 PMC5494561

[85] Schubert K Von Bonnsdorf H Burke M Ahlert I Braun S Berner R. A comprehensive candidate gene study on bronchial asthma and juvenile idiopathic arthritis. Dis Markers. (2006) 22:127–32. 10.1155/2006/373620 16788246 PMC3851125

[86] Heinzmann A Ahlert I Kurz T Berner R Deichmann KA. Association study suggests opposite effects of polymorphisms within IL8 on bronchial asthma and respiratory syncytial virus bronchiolitis. J Allergy Clini Immunol. (2004) 114:671–6. 10.1016/j.jaci.2004.06.038 15356575

[87] Lin C-H Lin C-L Shen T-C Wei C-C. Epidemiology and risk of juvenile idiopathic arthritis among children with allergic diseases: a nationwide population-based study. Pediat Rheumatol. (2016) 14:1–7. 10.1186/s12969-016-0074-8 26965056 PMC4787040

[88] Macaubas C Nguyen K Milojevic D Park JL Mellins ED. Oligoarticular and polyarticular JIA: epidemiology and pathogenesis. Nat Rev Rheumatol. (2009) 5:616–26. 10.1038/nrrheum.2009.209 19806151 PMC4159935

[89] Antonelli A Ferrari SM Giuggioli D Ferrannini E Ferri C Fallahi P. Chemokine (C–X–C motif) ligand (CXCL) 10 in autoimmune diseases. Autoimmun Rev. (2014) 13:272–80. 10.1016/j.autrev.2013.10.010 24189283

[90] de Jager W Hoppenreijs EP Wulffraat NM Wedderburn LR Kuis W Prakken BJ. Blood and synovial fluid cytokine signatures in patients with juvenile idiopathic arthritis: a cross-sectional study. Ann Rheum Dis. (2007) 66:589–98. 10.1136/ard.2006.061853 17170049 PMC1954617

[91] Rochette E Duché P Merlin E. Juvenile idiopathic arthritis and physical activity: possible inflammatory and immune modulation and tracks for interventions in young populations. Autoimmun Rev. (2015) 14:726–34. 10.1016/j.autrev.2015.04.007 25936296

[92] Butler A Van Lieshout RJ Lipman EL MacMillan HL Gonzalez A Gorter JW. Mental disorder in children with physical conditions: a pilot study. BMJ Open. (2018) 8:e019011. 10.1136/bmjopen-2017-019011 29301763 PMC5781020

[93] Fair DC Rodriguez M Knight AM Rubinstein TB. Depression and anxiety in patients with juvenile idiopathic arthritis: current insights and impact on quality of life, a systematic review. Open Access Rheumatol. (2019) 11:237–52. 10.2147/OARRR.S174408 31807093 PMC6830373

[94] Korte-Bouws GA Albers E Voskamp M Hendriksen H De Leeuw LR Güntürkün O. Juvenile arthritis patients suffering from chronic inflammation have increased activity of both IDO and GTP-CH1 pathways but decreased BH4 efficacy: implications for wellbeing, including fatigue, cognitive impairment, anxiety, and depression. Pharmaceuticals. (2019) 12:9. 10.3390/ph12010009 30625990 PMC6469185

[95] Reda MM Hosny E AbuSenna H. Psychiatric morbidity in patients with rheumatoid juvenile arthritis: a SPECT study. Middle East Curr Psychiat. (2011) 18:132–7. 10.1097/01.XME.0000398422.59986.78

[96] Lee H Jin Y Liu J Cohen EM Chen SK Kim SC. Risk of diabetes mellitus in patients with juvenile idiopathic arthritis. J Rheumatol. (2020) 47:1405–8. 10.3899/jrheum.190644 31787600

[97] Szabłowski M Okruszko MA Pochodowicz K Abramowicz P Konstantynowicz J Bossowski A. Coincidence of juvenile idiopathic arthritis and type 1 diabetes: a case-based review. Rheumatol Int. (2022) 42:371–8. 10.1007/s00296-021-05083-z 34999914 PMC8800897

[98] Koca B Sahin S Adrovic A Barut K Kasapcopur O. Cardiac involvement in juvenile idiopathic arthritis. Rheumatol Int. (2017) 37:137–42. 10.1007/s00296-016-3534-z 27417551

[99] Arsenaki E Georgakopoulos P Mitropoulou P Koutli E Thomas K Charakida M. Cardiovascular disease in juvenile idiopathic arthritis. Curr Vasc Pharmacol. (2020) 18:580–91. 10.2174/1570161118666200408121307 32268865

[100] Glerup M Rypdal V Arnstad ED Ekelund M Peltoniemi S Aalto K. Long-Term outcomes in juvenile idiopathic arthritis: eighteen years of Follow-Up in the Population-Based Nordic juvenile idiopathic arthritis cohort. Arthritis Care Res. (2020) 72:507–16. 10.1002/acr.23853 30762291

[101] Malleson PN Oen K Cabral DA Petty RE Rosenberg AM Cheang M. Predictors of pain in children with established juvenile rheumatoid arthritis. Arthritis Care Res. (2004) 51:222–7. 10.1002/art.20238 15077263

[102] Hersh AO Salimian PK Weitzman ER. Using patient-reported outcome measures to capture the patient's voice in research and care of juvenile idiopathic arthritis. Rheumatic Dis Clinics. (2016) 42:333–46. 10.1016/j.rdc.2016.01.004 27133493 PMC4853816

[103] De Lalouvière LLH Ioannou Y Fitzgerald M. Neural mechanisms underlying the pain of juvenile idiopathic arthritis. Nat Rev Rheumatol. (2014) 10:205–11. 10.1038/nrrheum.2014.4 24492386

[104] Walker SM. Overview of neurodevelopment and pain research, possible treatment targets. Best Pract Res Clini Rheumatol. (2014) 28:213–28. 10.1016/j.berh.2014.03.007 24974059

[105] Soulsby WD Balmuri N Cooley V Gerber LM Lawson E Goodman S. Social determinants of health influence disease activity and functional disability in Polyarticular Juvenile Idiopathic Arthritis. Pediat Rheumatol. (2022) 20:18. 10.1186/s12969-022-00676-9 35255941 PMC8903717

[106] Sullivan KE. Inflammation in juvenile idiopathic arthritis. Rheumatic Dis Clini North Am. (2007) 33:365–88. 10.1016/j.rdc.2007.07.004 17936170

[107] You L Qiu A Huang B Qiu P. Early detection of high disease activity in juvenile idiopathic arthritis by sequential monitoring of patients' health-related quality of life scores. Biom J. (2020) 62:1343–56. 10.1002/bimj.201900127 32159871

[108] Prahalad S Glass DN. A comprehensive review of the genetics of juvenile idiopathic arthritis. Pediat Rheumatol. (2008) 6:1–16. 10.1186/1546-0096-6-11 18644131 PMC2515830

[109] Rigante D Bosco A Esposito S. The etiology of juvenile idiopathic arthritis. Clin Rev Allergy Immunol. (2015) 49:253–61. 10.1007/s12016-014-8460-9 25384710

