# Supplementary material for: Development of a machine learning-based predictive nomogram for screening children with juvenile idiopathic arthritis: a pseudo-longitudinal study of 223,195 children in the United States
Source: Front Public Health. 2025 May 29;13:1531764. doi: 10.3389/fpubh.2025.1531764 (PMC12158982; doi:10.3389/fpubh.2025.1531764)
Supplement: Supplementary file 1 [file Table_1.docx]

Supplementary Table 1. Geographic distribution of the responders and children by State/district in NSCH data between 2016 and 2021

| **State/District** | **N** | **%** |
| --- | --- | --- |
| Alabama | 4,067 | 1.82 |
| Alaska | 3,981 | 1.78 |
| Arizona | 4,126 | 1.85 |
| Arkansas | 4,174 | 1.87 |
| California | 4,142 | 1.86 |
| Colorado | 5,959 | 2.67 |
| Connecticut | 4,306 | 1.93 |
| Delaware | 4,099 | 1.84 |
| District of Columbia | 4,116 | 1.84 |
| Florida | 4,139 | 1.85 |
| Georgia | 4,607 | 2.06 |
| Hawaii | 4,572 | 2.05 |
| Idaho | 4,247 | 1.90 |
| Illinois | 4,370 | 1.96 |
| Indiana | 4,129 | 1.85 |
| Iowa | 4,283 | 1.92 |
| Kansas | 4,318 | 1.93 |
| Kentucky | 4,129 | 1.85 |
| Louisiana | 4,067 | 1.82 |
| Maine | 4,148 | 1.86 |
| Maryland | 4,183 | 1.87 |
| Massachusetts | 4,370 | 1.96 |
| Michigan | 4,223 | 1.89 |
| Minnesota | 4,357 | 1.95 |
| Mississippi | 3,989 | 1.79 |
| Missouri | 4,279 | 1.92 |
| Montana | 4,344 | 1.95 |
| Nebraska | 4,817 | 2.16 |
| Nevada | 3,956 | 1.77 |
| New Hampshire | 4,254 | 1.91 |
| New Jersey | 4,325 | 1.94 |
| New Mexico | 3,965 | 1.78 |
| New York | 4,108 | 1.84 |
| North Carolina | 4,085 | 1.83 |
| North Dakota | 4,191 | 1.88 |
| Ohio | 4,814 | 2.16 |
| Oklahoma | 4,061 | 1.82 |
| Oregon | 8,593 | 3.85 |
| Pennsylvania | 4,194 | 1.88 |
| Rhode Island | 4,012 | 1.80 |
| South Carolina | 4,221 | 1.89 |
| South Dakota | 4,291 | 1.92 |
| Tennessee | 3,983 | 1.78 |
| Texas | 4,004 | 1.79 |
| Utah | 4,318 | 1.93 |
| Vermont | 4,364 | 1.96 |
| Virginia | 4,292 | 1.92 |
| Washington | 4,360 | 1.95 |
| West Virginia | 3,953 | 1.77 |
| Wisconsin | 6,440 | 2.89 |
| Wyoming | 3,870 | 1.73 |
| Total | 223,195 | 100 |

Supplementary Table 2. Comparisons of the LASSO logistic regression and logistic regression identified predictors for JIA by children with and without JIA information

|  | With JIA information | Without JIA information (missed) |  |
| --- | --- | --- | --- |
|  | N (%) | N (%) | p-value |
| **Child’s age when survey [Mean(SD)]** | 9.14 (5.26) | 10.00 (5.14) | <.0001* |
| **Sex** |  |  | 0.3008 |
| Boy | 115533 (51.8) | 1139 (50.7) |  |
| Girl | 107662 (48.2) | 1109 (49.3) |  |
| **Race** |  |  | <.0001* |
| Hispanic | 27533 (12.4) | 322 (14.4) |  |
| White, non-Hispanic | 152152 (68.3) | 1364 (60.9) |  |
| Black, non-Hispanic | 14071 (6.3) | 244 (10.9) |  |
| Asian, non-Hispanic | 11727 (5.3) | 164 (7.3) |  |
| American Indian or Alaska Native Non-Hispanic | 1331 (0.6) | 17 (0.8) |  |
| Others | 15847 (7.1) | 128 (5.7) |  |
| **Low birth weight** |  |  | 0.0208* |
| No | 195145 (91.6) | 1876 (91.2) |  |
| Low birth weight | 15180 (7.1) | 141 (6.8) |  |
| Very low birth weight | 2769 (1.3) | 41 (2.0) |  |
| **BMI** |  |  | <.0001* |
| Normal weight | 112564 (51.5) | 969 (45.3) |  |
| Children age 0-9 years, BMI not measured | 6699 (3.1) | 67 (3.1) |  |
| Underweight | 69398 (31.7) | 739 (34.6) |  |
| Overweight or obese | 30009 (13.7) | 362 (16.9) |  |
| **Child’s household food insecurity** |  |  | <.0001* |
| always afford to eat good nutritious meals | 163522 (74.9) | 1510 (70.2) |  |
| always afford enough to eat but not always the kinds of food we should eat | 47050 (21.6) | 548 (25.5) |  |
| Sometimes could not afford enough to eat | 6523 (3.0) | 74 (3.4) |  |
| Often could not afford enough to eat | 1182 (0.5) | 19 (0.9) |  |
| **Adequacy of current insurance coverage** |  |  | <.0001* |
| Adequate | 152264 (68.6) | 1459 (65.9) |  |
| Not adequate | 60051 (27.1) | 619 (28.0) |  |
| Uninsured | 9617 (4.3) | 136 (6.1) |  |
| **Children with a personal doctor or nurse (Yes)** | 170517 (76.8) | 1533 (69.8) | <.0001* |
| **Children’s health condition (Yes)** |  |  |  |
| Anxiety | 20078 (10.5) | 156 (8.1) | 0.0005* |
| Allergy to food, drug, or insect | 19661 (8.8) | 62 (3.1) | <.0001* |
| Asthma | 4845 (2.2) | 31 (1.6) | 0.0823 |
| Chronic physical pain in the past 12 months | 14219 (6.4) | 139 (6.9) | 0.3856 |
| Type 1 Diabetes | 909 (0.4) | 13 (0.7) | 0.0931 |
| Difficulty with eating or swallowing in the past 12 months | 3239 (1.5) | 26 (1.3) | 0.5190 |
| Genetic or inherited condition | 8538 (3.9) | 64 (2.9) | 0.0233* |
| Heart condition | 3068 (1.4) | 17 (0.8) | 0.0274* |

* p-value < 0.05
